# Open ankle fractures are associated with complications and reoperations

**DOI:** 10.1097/OI9.0000000000000042

**Published:** 2019-11-25

**Authors:** Natasha M. Simske, Megan A. Audet, Chang-Yeon Kim, Heather A. Vallier

**Affiliations:** MetroHealth Medical Center, Cleveland, Ohio, affiliated with Case Western Reserve University

**Keywords:** ankle fracture, complications, dislocation, elderly, fall, open fracture, outcomes

## Abstract

**Objectives:**

To assess clinical and functional outcomes after open versus closed ankle fracture.

**Design:**

Retrospective comparative study.

**Location:**

Level 1 Trauma Center.

**Patients/Participants:**

1303 patients treated for ankle fractures (Weber B and C) between 2003 and 2015. One hundred sixty-five patients (12.7%) presented with open fracture and 1138 (87.3%) with closed fracture.

**Intervention:**

Surgical or conservative management of ankle fracture.

**Main Outcome Measure:**

Rates of complications and reoperations. Patient-reported functional outcomes were assessed with the Foot Function Index (FFI) and Short Musculoskeletal Function Assessment (SMFA), after a minimum of 12 months.

**Results:**

Mean age was 46 years and 49% of patients were male. Higher mean age was associated with open injuries (51 vs 45 years, *P* < 0.001), and fractures were increasingly open with aging. Open fractures were associated with high-energy mechanisms: 44% following motor vehicle or motorcycle collisions, although the majority of open fractures in patients >65 years occurred after ground-level fall. Complications occurred more often after open fracture (33% vs 11%) and necessitated more secondary procedures (19% vs. 7%), both *P* < 0.001. Multivariate regression analysis identified open fracture as a predictor of complications and of worse scores on the activity categories of both the FFI and SMFA.

**Conclusion:**

Open fractures occurred more often after high energy mechanisms, and were generally more complex than closed fractures. Advanced age was common among open fracture patients, likely contributing to higher complication and secondary procedure rates. Greater morbidity after open ankle fractures was associated with minor differences on activity functions of the FFI and SMFA.

**Level of Evidence**: Level 3, prognostic

## 1 INTRODUCTION

Ankle fractures are common, and are treated by most orthopaedic surgeons, with a sizable proportion of these being open injuries.^[1–4]^ Among the elderly population, ankle fractures due to low-energy mechanisms are likewise increasing in prevalence.^[5–7]^ Ankle injuries are additionally common among athletes, where 15% to 25% of all athletic-related injuries occur at the ankle.^[8–10]^

Historically, research on ankle fractures has centered on surgical timing or technique, varying outcomes due to cigarette or alcohol consumption, comorbidities, or osteoporosis.^[4,11–18]^ The epidemiology of open versus closed ankle fractures has not been as broadly investigated. Specifically, there is sparse information regarding how injury characteristics associated with ankle fracture vary over the life course. Evidence regarding common patient characteristics that correlate with increased risk for poor outcomes and high complication rates would aid provider planning of tailored treatment options and encourage patient-directed care.

Prior study has often focused on the high-energy trauma that frequently leads to open ankle fracture versus relaying information on other common groups afflicted, namely the elderly who typically sustain ankle fractures via low-energy means.^[4–7]^ Studies investigating ankle fractures in the elderly population are often limited by small numbers of subjects identified retrospectively, making it difficult to draw meaningful conclusions.^[19]^ This paper will explore epidemiology, fracture patterns, and complication rates associated with open versus closed ankle fractures, while also categorizing common groups sustaining both types of ankle fracture.

## 2 PATIENTS AND METHODS

Following Institutional Review Board approval, a database at a level 1 trauma center was queried for patients with ankle fractures (AO/OTA 44B-C).^[20]^ 1303 skeletally mature patients were treated operatively or nonoperatively for such injuries between 2003 and 2015. Patients were subdivided based on whether ankle fracture was open (n = 165): Gustilo and Anderson Type 1: 8, Type 2: 29, Type 3: 128 with 119 3A, 8 3B and 1 3C, or if ankle fracture was closed (n = 1138). Patients were excluded from the study if medical records were missing or incomplete or if they sustained a Weber type A fracture.

### 2.1 Variables of interest

Charts and radiographs were reviewed for demographic data, including age, sex, body mass index (BMI), and presence of medical comorbidities such as diabetes mellitus, obesity, congestive heart failure, coronary artery disease, chronic obstructive pulmonary disease, autoimmune disease, peripheral vascular disease, and psychiatric illness. Tobacco, alcohol, and recreational drug use were defined as current or former use. Mechanism of injury, Weber and AO/OTA classifications, as well as other injury features were also noted. Timing of surgery was calculated based on time of injury and subsequent surgery date. Secondary procedures including elective implant removal were recorded. Patient-reported outcome measures (PROMs) were also obtained after minimum 12 months after injury using the Foot Function Index (FFI, n = 507) and Short Musculoskeletal Function Assessment (SMFA, n = 507).^[21–23]^ Patients were contacted to complete these surveys via phone or mail on up to 3 occasions by research staff not involved in clinical care.

### 2.2 Treatment

Ankle fractures were treated surgically using standard techniques of open reduction and internal fixation. Open fractures were treated with urgent surgical debridement followed by open reduction and internal fixation using small fragment and/or mini fragment stainless steel implants. Ten patients with open fractures underwent provisional external fixation and returned to the operating room at a later time for repeat debridement and fixation. Mean time to definitive surgery was 6.9 days. All patients were splinted postoperatively. Non-weightbearing and elevation were recommended initially, at the discretion of the treating physician. Based on fracture pattern and both clinical and radiographic observations, weightbearing was deferred for between 6 and 12 weeks. Complications were recorded, including superficial infection, deep infection, nonunion, and malunion. Infections were either superficial, treated on an outpatient basis with local wound care and oral antibiotics; or deep, requiring surgical debridement and intravenous antibiotics. Wound-healing complications including any wound draining, necrosis, or dehiscence that required additional wound care were likewise recorded. Malunions were described as >5° of tibiotalar or fibular angular deformity in any plane, based upon standing radiographs once weightbearing had been initiated. Nonunions were defined as lack of complete healing to any fracture component (lateral, medial, or posterior) within 6 months.

### 2.3 Statistical analysis

Independent sample *t* tests were used to compare means of continuous and ordinal variables, such as age and BMI, between patients sustaining open fractures versus those with a closed presentation. Two-tailed Fisher exact tests or Pearson chi-squared tests were utilized, depending on sample size, to compare categorical variables between patients with open fractures to those sustaining closed ankle fractures. Multivariate regression was performed to investigate relationships between clinical outcomes, including complications and between functional outcome scores (FFI and SMFA) and patient demographics (age, sex), medical history (obesity, diabetes, psychiatric illness, tobacco use), and injury features (pattern, open fracture, and history of dislocation). *P* values equal to or less than 0.05 were considered to represent statistical significance.

## 3 RESULTS

One thousand three hundred and three patients, 662 women (51%) and 641 men (49%), were included with mean age 46 years. Medical comorbidities were common, including 212 patients (15%) with diabetes mellitus and 216 (17%) with a psychiatric illness. Substance-use was common: 59% used tobacco products, 44% reported alcohol use, and 13% reported recreational drug use. Patients with open fractures (n = 165, 13%) were more likely to be older: 51 vs 45 years (*P* < 0.001). Patients with open versus closed fractures were no different in terms of obesity, diabetes, psychiatric illness, or reported substance use (Table 1).

**Table 1:**
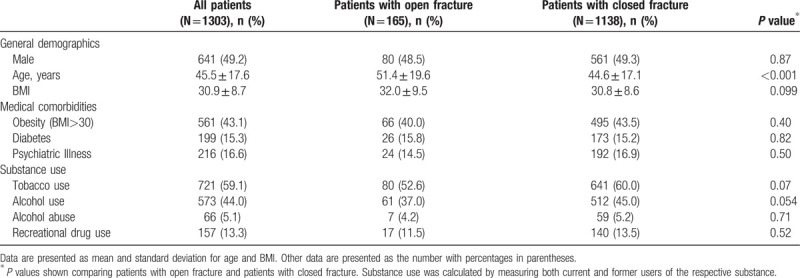
Patient demographics and social factors.

Closed fractures were associated with lower-energy mechanisms, such as ground-level falls: 36% of open fractures vs 67% of closed fractures (*P* < 0.001). Open fractures were often sustained after high-energy trauma including motor vehicle and motorcycle collisions (44% vs 20%) and crush injuries (5% vs1%), both *P* < 0.01. Open fractures were more often associated with ankle dislocation (73% vs 27%) and medial malleolus fractures (77% vs 55%), both *P* < 0.05. Closed ankle fractures were frequently isolated malleolar fractures (40% vs 20%), whereas open ankle fractures were more often bimalleolar fractures (52% vs 32%), both *P* < 0.001. Patients with open fractures had more associated injuries (38% vs 21%, *P* < 0.001). These findings are summarized in Table 2.

**Table 2:**
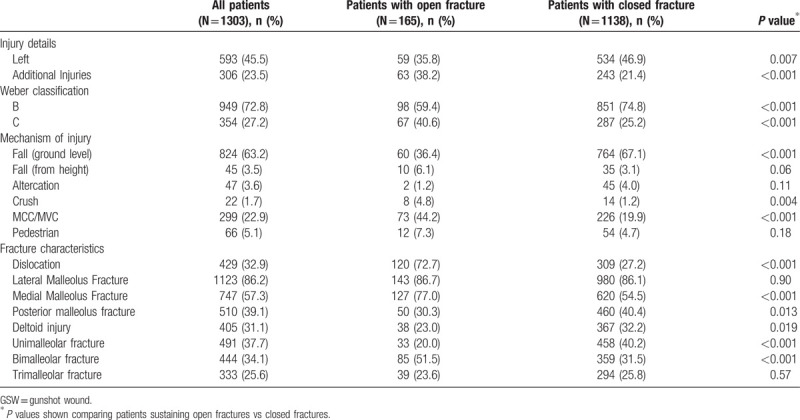
Injury characteristics are presented, including fracture pattern and features and mechanism of injury.

**Figure 1:**
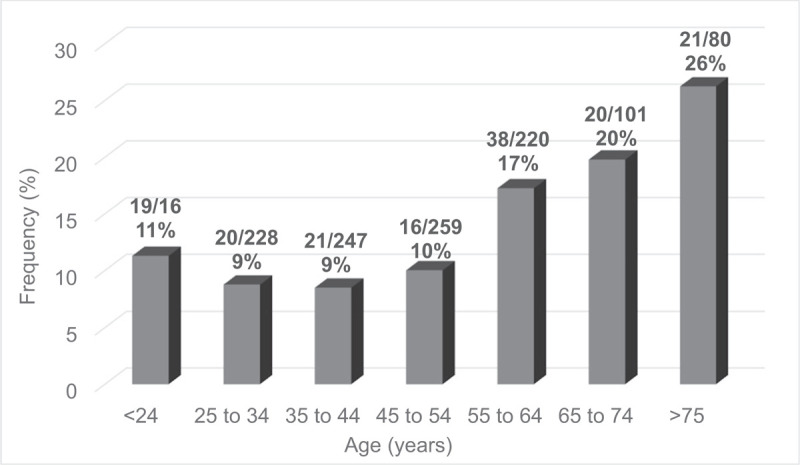
Prevalence of open ankle fracture over the life course.

**Figure 2:**
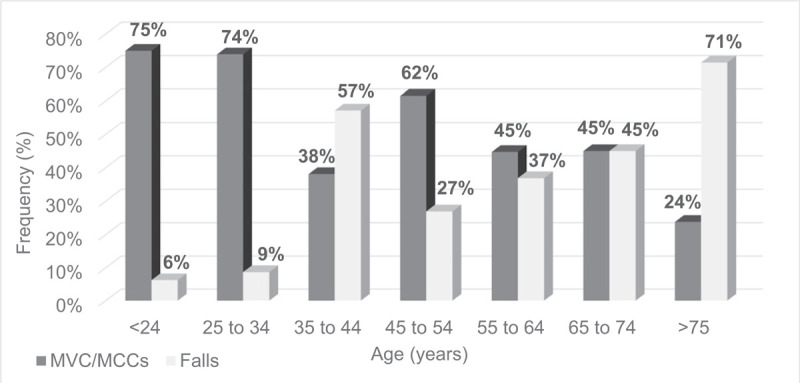
Mechanism of injury for open ankle fracture by age at time of injury.

Age corresponded to specific injury and fracture patterns. Incidence of open fractures peaked in patients aged 75 years or older, with 71% of these injuries attributable to ground level falls (Figs. 1 and 2). Twenty-three percent of ankle fractures (41/181) were open for persons older than 65. This number rose to 26% (21/80) for persons >75 years and continued to increase, as 29% (7/24) patients >85 years sustained open fractures. With aging, the majority of open fractures occurred after ground level falls (59% in ages >65 vs 29% in ages < 65, Table 3.

**Table 3:**
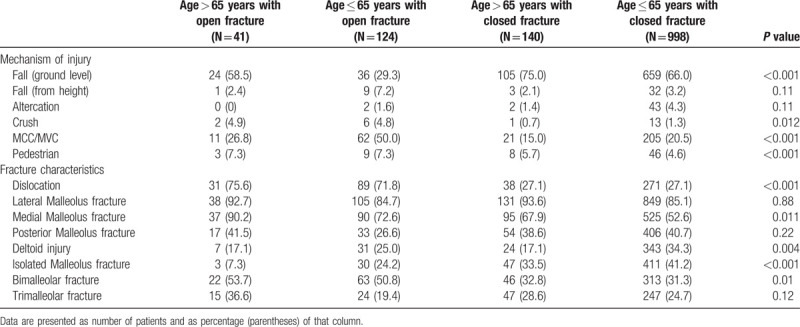
Mechanism of injury and fracture features are shown based on open versus closed fracture and by >65 versus less than or equal to 65 years

Complications developed in 178 patients (14%) and were associated with 111 additional procedures (8.5% of all patients); see Table 4. Complications occurred more after open ankle fractures (33% vs 11%, *P* < 0.001). Superficial infections, wound healing problems, delayed wound healing, and nonunion were all more common among open fracture patients (all *P*≤0.02). Secondary procedures were more common following open ankle fracture (19% vs 7%, *P* < 0.001). The open fracture population experienced higher rates of implant removal and amputation (both *P* < 0.05), while irrigation and debridement, implant revisions, and arthrodesis had similar occurrences. After multivariable analysis open fracture was most likely to be associated with developing a complication (*P* < 0.001), while presence of any medical comorbidity, including diabetes, tobacco, alcohol abuse, and/or renal disease, was also a risk factor (*P* = 0.001).

**Table 4:**
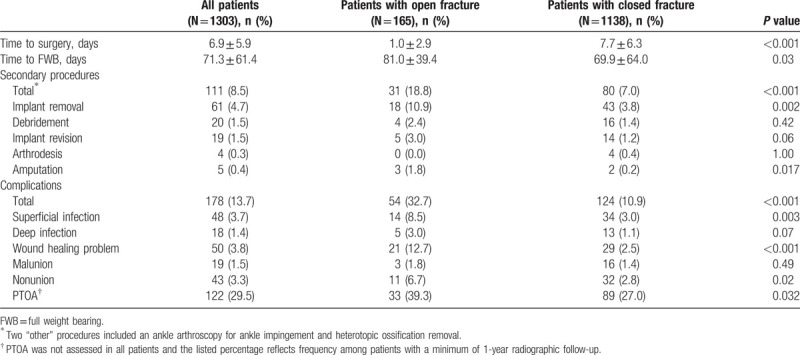
Complications after open versus closed ankle fracture.

FFI and SMFA scores were obtained after a mean 70 months following injury. Univariate comparison identified no significant differences among any of the individual indices, despite some worse subscores for the open fracture population. Activity subcategories in the FFI and SMFA were worse for persons sustaining open ankle fractures (FFI: 31 vs 24, *P* = 0.06 and SMFA: 33 vs 27, *P* = 0.08). Overall, the worst reported categories were the FFI's disability section (mean 38) and the SMFA's mobility section (mean 37.3). Full results are shown in Table 5. Multivariate regression analysis indicated that open fracture was predictive of poor FFI-activity scores (B = 7.62, *P* = 0.04) and suboptimal SMFA daily activity scores (B = 8.21, *P* = 0.037).

**Table 5:**
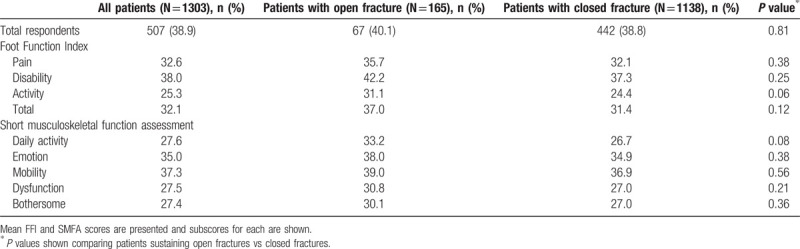
Patient-reported outcome measures.

## 4 DISCUSSION

Overall open ankle fractures comprise a small number of all ankle fractures, ranging from 1.5% to 7%.^[1,5,24,25]^ Some literature has reported an incidence of open ankle fractures as high as 18% to 29%, though allegedly this is due to oversampling of more severe trauma.^[3,26]^ We found 165 patients (12.7%) sustaining open ankle fractures, trending in the middle of these 2 spectra.

Mean age of patients sustaining ankle fractures ranges from 27 to 70 years among previous studies.^[1,5,19,24,26–28]^ Variability in mean age could depend on the number of open fractures compared to closed, or if certain subgroups were preferentially sampled, based on evidence that patients aged 10 to 19 and over 80 years sustain high rates of open and closed ankle fractures.^[1,28,29]^ In our study, we observed that patients aged 75 years or older had the highest frequency of open fracture (26%) and this finding increased to 29% in patients aged 85 years or older. This indicates a general trend where elderly patients sustain more ankle fractures that are open. Our increasingly aging population may transform typical patient demographics associated with ankle fracture. Although this has the potential to impact outcomes, Bray et al^[30]^ found no significant correlations between age and functional results after open ankle fracture.

Mechanism of injury is highly predictive of associated fracture characteristics. According to more recent literature, motor vehicle crashes (MVC) and motorcycle crashes (MCC) accounted for 20% and 26% of open ankle fractures.^[1,5]^ A study completed 25 years prior observed that MVCs comprised 64% of open ankle fractures and gunshot wounds accounted for 10%.^[29]^ Although substantial shifting in population demographics could explain some of these differences, the small sample set (n = 31) may skew toward sampling more severe trauma.^[30]^ Our study indicates a greater frequency of high-energy trauma, with road traffic collisions contributing to 43% of open ankle fractures. Based on prior study, closed fractures appear to have different injury patterns, with road traffic collisions comprising 9% to 53%, while low-energy mechanisms contribute to as many as 64% of these fractures.^[25,26,31]^ Low-energy falls similarly constituted 67% of our closed ankle fracture presentations.

Minimal prior work has investigated the relationship of age to mechanism of injury and presence of open injury. Bugler et al^[1]^ found in their elderly patient population that 74% of fractures could be attributed to simple falls. Our study supports this finding. In patients 65 years or older, 59% of open fractures and 75% of closed fractures were due to a ground level fall. We hypothesized that open ankle fractures would fall into 2 distinct patient subgroups and a bimodal age distribution: elderly persons with injuries due to low-energy falls and younger individuals who sustain injuries during high-energy trauma. Our results support this. High-energy mechanisms such as road collisions comprise the overwhelming majority of open fractures in young adults. This reverses during the life course to 71% of open ankle fractures being sustained following a ground level fall in patients 75 years or older.

Existing literature frequently reports fracture characteristics. Open fractures are unimalleolar 9% to 17% of the time, bimalleolar 38% to 55%, and trimalleolar 36% to 45%.^[5,28]^ Our findings are comparable, with 20% being unimalleolar, 52% being bimalleolar, and 24% being trimalleolar. These rates differ from those for closed ankle fractures, which are more frequently unimalleolar.^[28]^ In a cohort of 112 closed ankle fractures, 64 (57%) had a corresponding dislocation.^[31]^ We observed much lower rates, with 27% of closed fractures having an associated dislocation injury. Two groups reported that lateral ankle fractures are more common than medial among both mixed fracture and closed fracture populations.^[25,28]^ These results are analogous to our findings. Yet, we are unable to compare our findings in the open fracture population due to lack of similar studies. At this time, we find that lateral and medial malleolus fractures are common in the open fracture population, with medial malleolus fracture occurring more often with open fractures. Posterior malleolus fractures are comparatively uncommon in open fracture populations.

Complications are often discussed in relation to surgeon-specific practices such as timing of surgery,^[4,11–13,32]^ soft tissue handling,^[33]^ and implant type.^[34,35]^ The impact of patient demographics on complication rates is less frequently examined.^[35]^ Malunion, nonunion, implant failure, and wound-healing complications can occur after operative treatment of ankle fractures.^[36]^ Wound complications prevail most often and are impacted by patient age or comorbidities. For example, there is evidence indicating that diabetic patients have more postoperative complications such as deep infections or loss of fixation.^[4,27,37,38]^ Our study supports this finding, as patients with diabetes were more likely to have complications (32.5% vs 11.4%, *P* < 0.001). Furthermore, having diabetes was a factor associated with additional operations: 28 patients with diabetes (13.2%) required a second operation vs 85 patients (7.3%) without diabetes (*P* = 0.016). This probably occurs due to increased risk of inadequate soft-tissue and fracture healing after surgery in diabetic patients.^[36,39,40]^

Open fractures are twice as likely to lead to complications,^[27]^ thereby contributing to worse functional outcomes and greater disability than is the case with closed fractures.^[16,41,42]^ Our study supports this conclusion: 33% of open fracture patients developed a complication, compared with 11% among closed fractures. Furthermore, patients with open fracture required more implant removals and revisions. As our open fracture cohort had unplanned secondary operations 19% of the time, the trend toward greater morbidity and higher costs represents a tangible problem. Additionally, the routine care of an uncomplicated open ankle fracture would be expected to be greater than that of a closed fracture, since closed fractures would be treated on an outpatient basis, whereas open fractures would be admitted to the hospital for perioperative intravenous antibiotics.^[43,44]^

Worse functional outcomes among open ankle fractures are reportedly associated with complications.^[27]^ Our findings, however, do not readily support this. Neither overall scores, nor individual subcategory scores on the FFI or SMFA, were significantly different, despite clinically worse scores on the activity subgroups of both PROMs (Table 5). However, we identified trends toward worse activity subscores after open fracture with both instruments, as indicated by regression analysis. This is potentially reflective of more open fractures among elderly patients with requirement for ambulatory aids at baseline and/or following injury. Our findings are not attributable to sampling bias, as patients with either fracture type were just as likely to respond to the questionnaire. Given the mounting popularity of PROMs, there are few comparative studies. Egol et al^[42]^ found 3-month postoperative scores to be 22.6 on the dysfunction index of the SMFA and 24.3 on the bothersome index. Subsequently, these dropped back to normal or expected levels by 1 year. Our patients were assessed on average 70 months following their operative procedures and still reported higher mean scores. Other studies were unable to be used for comparison, as they both utilized the Olerud Molander Ankle Score (OMAS) as their index of choice.^[45,46]^

The primary strength of this study is the number of patient records that were reviewed, allowing for a substantial open fracture population (n = 165). The foremost limitation of this study is its retrospective nature, contributing to lack of recorded data on certain demographics and injury characteristics. The retrospective nature also limited our ability to obtain functional outcomes on the FFI and SMFA surveys from the entire study population, introducing a possible sampling bias whereby persons with greater pain and disability were more likely to respond to surveys. The authors do not believe this introduced a substantial problem, as response rates were similar between open and closed groups (40% vs 39%, *P* = 0.81). Similarly, patients were assessed for posttraumatic arthritis after minimum 12 months, unless such findings were present prior to that time. This limited the number of patients assessed for posttraumatic osteoarthritis (PTOA), and likely inflated our rate of PTOA. Advanced imaging was not obtained, thus quality of articular reduction and its possible relationship to PTOA could not be accurately assessed. Finally, our study observed a higher complication rate (13.7%) when compared to large database studies that report complications from 2% to 4%.^[47,48]^ This disparity is most likely a function of our population, inclusive of more high energy and open injuries, and possibly patients with more medical comorbidities. Furthermore, the nature of retrospective chart review lends itself to being more inclusive to minor complications (e.g. superficial infections and wound complications), that might not have received an international classification of diseases code had it been entered into such a database.

Our study presents evidence that open fractures predominate between 2 distinct populations: young adults injured in high-energy trauma and geriatric patients after low-energy falls. Furthermore, the highest prevalence of open fracture was observed in patients 75 years and older. With population aging, it is possible that more open ankle fractures will be seen among the elderly who sustain their injuries after a ground-level fall. This may lead to changes in treatment. Further identification of improved treatment options for low-energy geriatric ankle fractures may be beneficial, given the shifting paradigm. Identifying patients predisposed to higher complication rates and lengthened periods of hospitalization may help mitigate costs, improve outcomes, and enhance patient satisfaction.
